# Adolescents’ Preferred and Inferred Strategies for Being Accurately Understood by Their Parents

**DOI:** 10.1007/s10964-025-02193-w

**Published:** 2025-05-12

**Authors:** Hagit Sabato, Shay Gozlan, Tal Eyal

**Affiliations:** 1 The Hebrew University of Jerusalem, Jerusalem, Israel; 2 Ben-Gurion University of the Negev, Beer-Sheva, Israel

**Keywords:** Mind perception, Perspective-taking, Perspective-getting, Perceived understanding, Adolescent-parent relationship quality, Adolescents’ life satisfaction

## Abstract

Perceived understanding in close relationships has been shown to positively correlate with relationship satisfaction and well-being. Less is known, however, about the preferred means for promoting such perceptions. The current study investigated the strategies adolescents prefer their parents use for understanding their feelings, and whether these preferences match the strategies adolescents infer their parents use and the strategies the parents report using. In addition, the study examined whether these preferences, inferences, and actual (reported) strategies correlate with the adolescents’ perceived understanding, life satisfaction, and relationship quality with their parents. The sample included 150 pairs of adolescents (M_age_ = 16.26; SD = 1.01, 57.3% female) and one of their parents (84% mothers, Mage = 47.94, SD = 6.51). The results revealed that adolescents mostly preferred their parents try to take their perspective (perspective-taking), whereas their parents reported using direct communication, that is, asking them about their feelings (perspective-getting). Perceived understanding, life satisfaction, and relationship quality were not predicted by the parent’s reported behavior but rather were positively associated with the adolescent’s preferred and inferred perspective-getting and negatively associated with the adolescent’s preferred perspective-taking.

## Introduction

Perceived understanding—people’s perceptions of the degree to which others accurately understand their feelings and thoughts—plays a critical role in relationships. It has been found to positively correlate with trust, subjective well-being and relationship satisfaction (even more than the perceiver’s actual understanding, Cohen et al., 2012; De Jong & Reis, 2014), and to negatively correlate with negative affect and conflict (Gordon & Chen, 2016; Gordon & Diamond, 2023; Hinnekens et al., 2019). While perceived understanding is particularly crucial in adolescent-parent relationship—in which communication is often a challenge (McLaren & Sillars, 2020)—research has yet to examine the means that best foster it. This study aimed to explore the strategies adolescents prefer their parents would use to accurately understand their feelings, and whether these preferences match the strategies adolescents infer their parents use and the strategies the parents report using. Furthermore, the study examined whether these preferred, inferred, and actual (reported) strategies were associated with adolescents’ perceived understanding, life satisfaction, and relationship quality with their parents.

Adolescence is a period of transmission, characterized by increased parent-child conflict, especially with regards to the adolescents’ need for independence and autonomy (Koepke & Denissen, 2012). It is often accompanied by increased distancing from the parents and decreased self-disclosure (Dotterer & Day, 2019). At the same time, adolescents and parents share core beliefs and values (e.g., Moskvicheva et al., 2016), most of them have close bonds and strong intimacy (e.g., Hochgraf et al., 2021‏), and adolescents tend to see their parents as significant role models (Hurd et al., 2009). Thus, although adolescents’ wish to be understood by their parents seems natural, this desire may conflict with their increased need for autonomy and separation.

Adolescents’ perception of their parents’ understanding plays an important role in their socioemotional functioning (Xiao et al., 2011). Parents’ warmth, including their efforts to understand their adolescent’s perspective, was found to be positively linked to adolescents’ feelings of being loved (Coffey et al., 2022). In addition, parents’ accurate understanding of their adolescent’s self-perception positively correlated with greater parent-adolescent relationship satisfaction (Sillars et al., 2005). More broadly, feeling understood by supportive adults was found to be related to better well-being, mental health, and help-seeking behavior among young people (aged 16–24), whereas feeling misunderstood had the opposite effect (Cunningham et al., 2024).

### Strategies for Accurate Understanding

Given that perceived understanding is at the heart of constructive communication and satisfaction in close relationships (Reis et al., 2017), identifying factors that may promote it is important. Research among adults has identified several strategies people commonly use to understand others’ minds. One common strategy for understanding others is to rely on the perceiver’s own mind (i.e., *projection*). Such egocentric inferences enable insight when one’s perspective is similar to another’s, but they can lead to misunderstanding when perspectives diverge (Epley & Eyal, 2019). Shifting one’s perspective to the target’s perspective (i.e., *perspective taking*) has been found to be helpful in overcoming egocentric biases, but not in reliably increasing accurate understanding (Eyal et al., 2018).

Another common source for inferences about others’ minds is theories people form about them (Gopnik, 2003), based on their *previous knowledge* about the target and on the target’s *observed behavior*. These knowledge-based strategies may lead to some degree of understanding but also to error because people’s memory is limited (Macrae & MacLeod, 1999) and because they assume others’ behavior reflects their mental states, neglecting the context in which the behavior occurs (Aviezer et al., 2012). Because the inferences these strategies are based on are typically biased, and partial, accurate understanding often requires a strategy that bypasses inferences altogether, by receiving individuating information from the target about their mental states (i.e., *perspective-getting*). Indeed, in a series of studies, participants who were guided to ask their partner about their preferences were more accurate in their predictions regarding the partner’s preferences compared with participants who were instructed to use perspective-taking, or control participants who received no instructions (Eyal et al., 2018). The benefit of perspective-getting over perspective-taking for accurate understanding has been replicated across different mental states (e.g., attitudes, preferences, traits) and dyads (e.g., friends, strangers, ideological opponents; Eyal et al., 2025).

Perceivers often fail to recognize which strategies are more versus less effective for gaining accurate understanding of others’ minds. For example, although perspective taking and observed behavior have been shown to be typically ineffective for accurate understanding, participants anticipated using them would increase mind-perception accuracy (Eyal et al., 2018; Zhou et al., 2017). In addition, although perspective getting has been found to promote accurate understanding, when perceivers were allowed to ask targets about as many items of a survey measuring either preferences, traits, or attitudes as they wished in order to accurately predict the targets’ ratings on the survey, perceivers asked about only ~50% of the items, leading to lower accuracy than the accuracy of those who were instructed to ask about all items of the survey (Eyal et al., 2025).

## Current Study

Whereas research on mind perception accuracy has mainly focused on adults trying to understand the minds of others (i.e., *mind perceivers)*, the current research focuses on what enables mind-perception accuracy from the perspective of the *targets*. The study examined the strategies adolescents prefer their parents use when trying to accurately understand their feelings, the adolescents’ inferences regarding the strategies their parents use when attempting to understand their feelings (i.e., inferred strategies) and the strategies the parents report using when attempting to understand them (i.e., actual strategies). The study also examined whether mismatches between the preferred, inferred, and actual strategies are linked to adolescents’ perceived understanding, the quality of their relationship with their parents, and their life satisfaction. Finally, the reasons adolescents attribute to their preferences for specific mind-perception strategies were measured. Because the current study was the first to examine strategies that adolescents prefer their parents use when attempting to understand them, several questions (all preregistered; see the Method section) rather than specific directional hypotheses were tested. The specific questions aimed to examine which strategies adolescents prefer their parents use when trying to understand how they feel, whether these preferred strategies differ from those adolescents infer their parents use and those parents report using, and whether these preferred, inferred, and actual strategies are related to perceived understanding, relationship satisfaction, and life satisfaction.

## Method

### Participants

One hundred fifty pairs of adolescents (57.3% female, M_age_ = 16.26; SD = 1.01) and one of their parents (84% mothers, M_age_ = 47.94, SD = 6.51) took part in the study, in return for a gift card of NIS 50 (approximately US$13). Adolescents aged 15–18 and their parents were recruited via social media using a snowball sampling method, primarily through parents’ groups on Facebook and WhatsApp. They were asked to complete an online questionnaire. It was clarified to the participants that participation in the study, which rewards compensation, requires both the adolescent and the parent to complete the research questionnaire in full. Therefore, all dyads included in the study are those in which both the parent and the child answered all the questions, with no missing data. Data from additional 16 adolescents and 32 parents were excluded due to the absence of a corresponding partner (i.e., a parent or an adolescent) or incomplete questionnaires.

Data collection was conducted from November 2022 to July 2023. Sample size was determined based on similar studies (e.g., Goldberg, 2025). Sensitivity power analysis, conducted with G*Power (Faul et al., 2009) for regression analysis, indicated that, given N = 150, α = 0.05, and a power of 0.85, statistical significance would be detected with a small effect size (f^2^ = 0.106, critical f = 2.16).

Data, hypotheses, methods, and analyses were preregistered and are available at https://osf.io/863ex/.

The study received approval from the authors’ university ethics committee (#267-1).

### Procedure and Measurements

Both the parent and the adolescent first signed a consent form and read they could withdraw from the study at any time. Parents completed the questionnaire first. At the end of the questionnaire, they provided their child’s phone number, to which a link to the child’s online questionnaire was sent. Throughout the survey, the text that adolescents read referred to the parent’s gender (mother or father) in accordance to the parent who answered the parents’ questionnaire.

#### Adolescents’ Questionnaire

At the outset of the questionnaire, the adolescents read, “We all experience situations in which we are in a bad mood. Please recall a situation, in which you felt moody or upset about something that happened with friends, at school, or somewhere else, and for which you wanted your parent to understand exactly how you felt.” Next, they were presented, in random order, with five common strategies for accurately predicting others’ mental states (Epley & Eyal, 2019):

*Perspective-taking*: “I wish my parent would take my point of view, putting themselves in my shoes, as if they were me.”

*Perspective-getting*: “I wish my parent would ask me how I feel.”

*Projection:* “I wish my parent would think how they feel at that moment.”

*Prior knowledge*: “I wish my parent would use what they know about me. For example, what I like, past experiences, my traits, and the way I usually behave.”

*Observed behavior*: “I wish my parent would read my body language and observe my behavior.”

Participants were asked to (1) rate the extent to which they wanted their parent to use each strategy to accurately understand their feelings (hereafter, “preferred strategy”), (2) choose the one strategy they would prefer their parent use the most (hereafter “choice of preferred strategy”), and (3) rate three motivations for choosing that strategy (i.e., “the strategy helps my parent accurately understand my feelings,” “the strategy allows good communication between me and my parent,” “the strategy shows my parent cares about me”), or provide another explanation for their choice. Then, they rated the extent to which they thought their parent actually uses each strategy (hereafter “inferred strategy”) and chose the strategy they thought their parent uses the most (hereafter “choice of inferred strategy”). Participants then rated the extent to which they felt their parent accurately understands their feelings, in general (hereafter “perceived understanding”). All ratings were provided on a 7-point scale (1 = *not at all*, 7 = *very much*). Finally, participants completed several measures:

*Relationship Quality*: a four-item questionnaire (Johnson & Galambos, 2014) that assesses the quality of the relationship between the adolescent and the parent on a 7-point scale (1 = *strongly disagree*, 7 = *strongly agree*). The four items refer to the extent to which the relationship feels supportive and warm: “I feel close to my parent”; “Most of the time, my parent is warm and loving toward me”; “I am satisfied with the way my parent communicate with me” and; “Overall, I am satisfied with my relationship with my parent.”

*Life Satisfaction*: *Satisfaction with Life Scale* (SWLS—Diener et al., 1985) consists of five items (e.g., “In most ways my life is close to the way I would want it to be”), adapted for children and teens (ages 10 and above, SWLS-C, Gadermann et al., 2010) on a 5-point scale (1 = *strongly disagree*, 5 = *strongly agree*). Because life satisfaction is a more cognitive aspect of well-being (Diener, 2000), positive and negative feelings were also measured (SPANE, Diener, 2009), to be included as a covariate in the analyses.

#### Parents’ Questionnaire

Parents first wrote the name and gender of their child, and this information allowed the questions to be presented in a personalized manner. Then, the parent read:

“We all experience situations in which we are in a bad mood. Please recall a situation in which your child [the name of the child was presented according to the parent’s answer, as described above] felt moody or upset about something that happened with friends, at school, or somewhere else, and you wanted to understand accurately how they felt.”

Next, the parents read about the same five strategies from the adolescents’ questionnaire:

*Perspective-taking*: “ I usually try to take my child’s point of view, putting myself in their shoes, as if I were them.”

*Perspective-getting*: “I usually ask them how they feel.”

*Projection:* “I usually use the feeling I experience myself that moment.”

*Prior knowledge*: “I usually use what I know about my child. For example, what they like, past experience, their traits and the way they usually behave.”

*Observed behavior*: “I usually read my child’s body language and observe their behavior.”

Participants first rated the extent to which they use each strategy to understand their child’s feelings (hereafter “actual strategy”) and then chose the strategy they use the most (hereafter “choice of actual strategy”). Next, they rated the three motivations guiding their choice of the most reported strategy, the extent to which they think they accurately understand their child’s feelings, and the quality of the relationship with their child (Johnson & Galambos, 2014, the parent version, e.g., “overall, I am satisfied with my relationship with my child”). All ratings were provided on a 7-point scale (1 = *not at all*, 7 = *very much*). Finally, participants answered demographic questions and provided the adolescent’s phone number, which was used to deliver the payment and match the parent’s and child’s questionnaires.

## Results

### Ratings of Preferred, Inferred, and Actual Strategies

A repeated-measures ANOVA with strategy (perspective-taking, perspective-getting, previous knowledge, observed behavior, projection) and perception (preferred, inferred, actual) as within-subjects factors revealed a significant main effect for strategy, *F*(4, 144) = 33.87, *p* < 0.001*, η*_p_^2^ = 0.485, such that perspective-getting ratings were significantly lower than ratings of all other strategies (*p* < 0.001), and perspective-taking ratings were overall lower than ratings of all other strategies (*p* < 0.001), except for perspective-getting. The main effect of perception was also significant, *F*(2, 146) = 63.45, *p* < 0.001*, η*_p_^2^ = 0.465. Post hoc comparisons revealed that parents’ ratings of the actual use of strategies (*M* = 5.22, *SD* = 0.77) were higher than adolescents’ ratings of preferences (*M* = 4.90, *SD* = 1.11), *p* < 0.001, and inferences (*M* = 4.66, *SD* = 0.91), *p* < 0.001, and preferred strategies’ ratings were significantly higher than inferred strategies’ ratings, *p* = 0.002.

**Fig. 1:**
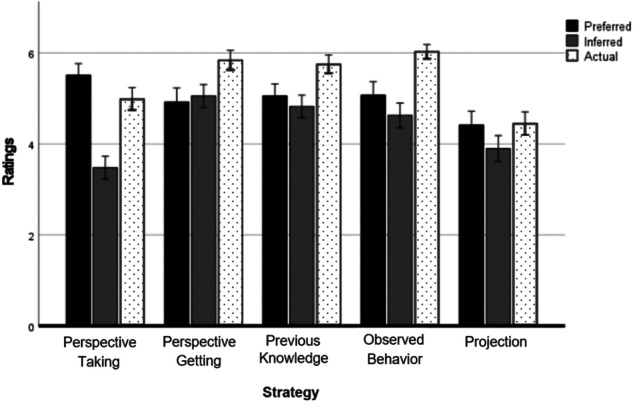
Ratings of the five strategies as a function of the strategy’s perception (preferred, inferred, and actual). Note: Error bars reflect 95% CI

The interaction between strategy and context was also significant, *F*(8, 140) = 12.91, *p* < 0.001*, η*_p_^2^ = 0.425. Post hoc comparisons revealed that preferred perspective-taking was significantly higher than all other preferred strategies, whereas inferred perspective-taking was significantly lower than all other strategies. Observed behavior, on the other hand, was significantly higher than other strategies in the parents’ ratings of actually used strategy, although adolescents preferred and inferred it less. Ratings of preferred, inferred, and actual ratings of the five strategies are presented in Fig. 1. The detailed comparisons are fully reported in the Supplementary Materials.

### Choice of Preferred, Inferred, and Actual Strategies

**Fig. 2:**
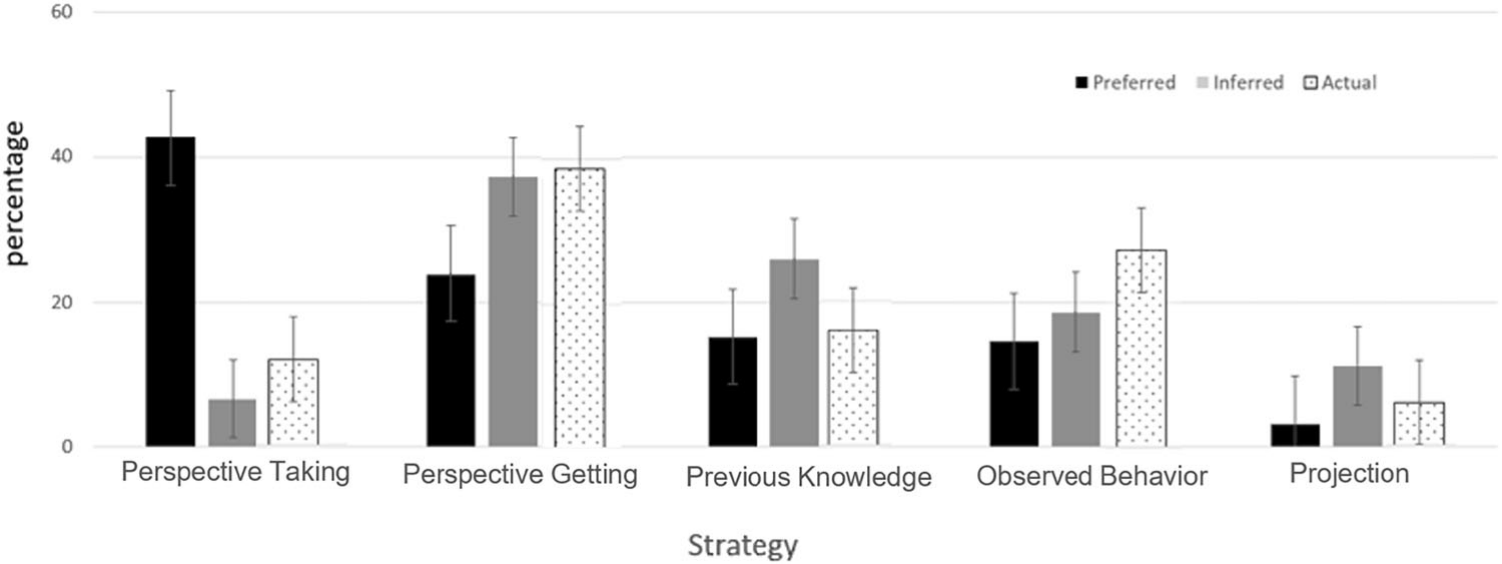
Strategies adolescents chose as the most preferred and inferred, and parents as most actually used. Note: Error bars reflect 95% CI

As Fig. 2 shows, adolescents mostly preferred their parents use perspective-taking (42.7%), followed by perspective-getting (24%), and mostly inferred their parents use perspective-getting (37.3%) and previous knowledge (26%). Parents mostly reported using perspective-getting (38.7%) and observed behavior (27.3%).

A test of non-parametric-pairs samples (Wilcoxon) revealed that parents’ and children’s preferences were overall significantly different (*z* = 5.645 *p* < 0.001). Only in 29.3% (44 out of 150) of the cases a match was found between the adolescent’s choice of preferred strategy and the parent’s choice of actual strategy.

To better understand the role of the two most preferred strategies, perspective-taking and perspective-getting, the same test of non-parametric-pairs samples (Wilcoxon) was conducted to compare adolescents’ and parents’ choices for each strategy. A dichotomous variable was computed for the “choice of preferred strategy” and for the “choice of actual strategy,” focusing on perspective-taking (coded 1; all other choices were coded 0), and another dichotomous variable was conducted for the “choice of preferred strategy and “choice of actual” strategy, focusing on perspective-getting (coded 1; all other choices were coded 0).

For perspective-taking, the difference between the most preferred strategy and the most used strategy was significant, *z* = 5.66, *p* < 0.001. Whereas 42.7% of the adolescents chose perspective-taking as their most preferred strategy, only 12% of parents reported actually using this strategy. By contrast, 38.7% of the parents chose perspective-getting as the strategy they used the most, whereas only 24% of the adolescents chose it as their most preferred strategy, *z* = 2.94, *p* = 0.003.

### Adolescents’ Motivations Underlying Their Choice of Preferred Strategy

To explore the motivations underlying adolescents’ choice of their most preferred strategy, a repeated-measures ANOVA on the three motivations as a within-subject factor was conducted, for each strategy separately. Descriptive statistics for the three motivations in each strategy (except for projection, which only five participants chose) are presented in Table 1. Ratings differed across the motivations in two of the strategies. For perspective-getting, the analysis revealed a significant effect of motivation (*p* = 0.022), such that adolescents rated good communication higher than showing care (*p* = 0.019) (the comparison to accurate understanding was marginally significant, *p* = 0.08). A significant effect was also found for previous knowledge (*p* = 0.020): accurate understanding was rated significantly lower than good communication (*p* = 0.014) and showing care (*p* = 0.021).

**Table 1:** Descriptive statistics of the three motivations, for each strategy choice (Adolescents)

	Accurate understanding	Good communication	Show care
Perspective-Taking (N = 64)	5.88^a^ (1.05)	5.42^a^ (1.41)	5.53^a^ (1.70)
Perspective-Getting (N = 36)	5.92^a,b^ (1.36)	6.33^a^ (1.12)	5.64^b^ (1.84)
Previous Knowledge (N = 23)	6.40^a^ (0.89)	5.40^b^ (1.95)	5.20^b^ (1.48)
Observed Behavior (N = 22)	4.68^a^ (1.94)	4.82^a^ (1.60)	4.95^a^ (2.03)
Total (N = 150)	5.53^a^ (1.47)	5.56^a^ (1.42)	5.43^a^ (1.80)

Note. Within each row, numbers that do not share a superscript (e.g., a, b) differ significantly at p < 0.05

### Parents’ Motivations for their Choice of Actual Strategy

To explore the motivations underlying parents’ choice of the strategy they mostly use, a repeated-measures ANOVA on the three motivations as a within-subject factor was conducted, for each strategy separately. Descriptive statistics for the three motivations in each strategy (except projection, which only nine participants chose) are presented in Table 2. Ratings differed across the motivations for three strategies:

**Table 2:** Descriptive statistics of the three motivations, for each strategy choice (parents)

	Accurate understanding	Good communication	Show care
Perspective -Taking (N = 18)	5.61^a^ (1.14)	5.72^a^ (1.41)	5.67^a^ (1.81)
Perspective -Getting (N = 58)	5.55^a^ (1.42)	6.02^b^ (1.33)	5.66^a,b^ (1.64)
Previous Knowledge (N = 24)	4.96^a,b^ (1.33)	5.42^b^ (1.41)	4.71^a^ (1.68)
Observed Behavior (N = 41)	5.46^a^ (1.18)	4.51^b^ (1.55)	4.73^b^ (2.03)
Total (N = 150)	5.39^a^ (1.38)	5.40^a^ (1.56)	5.21^a^ (1.74)

Note. Within each row, numbers that do not share a superscript (e.g., a, b) differ significantly at p < 0.05

The analysis revealed a significant effect of motivation for perspective-getting (*p* = 0.006). Ratings of good communication were significantly higher than accurate understanding (*p* = 0.009). There was also a significant effect for previous knowledge (*p* = 0.035): Parents rated good communication higher than showing care (*p* = 0.04) and accurate understanding (*p* = 0.053). Finally, for observed behavior, the effect was also significant (*p* < 0.001). Accurate understanding was rated higher than good communication (*p* < 0.001) and showing care (*p* = 0.025).

Next, it was examined whether preferred, inferred, and actual strategy and the interactions between them predicted adolescents’ perceived understanding, relationship quality with their parents, and life satisfaction. The focus was on perspective-taking and perspective-getting, the two most preferred strategies. The analyses for observed behavior, previous knowledge, and projection are reported in the Supplementary Materials.

In the following analyses, the same hierarchical regression model was used, for each strategy (perspective-taking, perspective-getting) separately. Alpha was corrected for multiple comparisons using the Benjamini-Hochberg adjustment (Benjamini & Hochberg, 1995). The three main effects (preferred, inferred, and actual) were entered in the first step and all two-way interactions in the second step. The detailed results are reported in Table 3. Interaction effects were interpreted using Hayes’ PROCESS Macro for SPSS (Hayes, 2013) Model 1. The full analyses are reported in Supplementary Materials.

**Table 3:** Linear hierarchical regression analyses for variables associated with relationship quality from the adolescent’s perspective (RQ-A), the adolescent’s life satisfaction, perceived understanding, for perspective-taking and perspective-getting

	B	SE	β	t	BHp
**Perceived Understanding**					
**Perspective Taking** Step 1					
1. Preferred Ratings	−0.093	0.074	−0.095	−1.249	0.214
2. Inferred Ratings	0.378	0.076	0.383	5.002	<0.001
3. Actual Ratings	0.112	0.077	0.111	1.457	0.441
Step 2					
1. Preferred X Inferred	0.162	0.048	1.142	3.334	0.003
2. Preferred X Actual	0.041	0.042	0.312	0.980	0.470
3. Actual X Inferred	−0.027	0.047	−0.176	−0.578	0.564
**Perspective Getting** Step 1					
1. Preferred Ratings	0.194	0.059	0.238	3.264	0.003
2. Inferred Ratings	0.370	0.072	0.378	5.173	<0.001
3. Actual Ratings	0.166	0.084	0.145	1.977	0.075
Step 2					
1. Preferred X Inferred	0.031	0.036	0.251	0.873	0.757
2. Preferred X Actual	−0.058	0.048	−0.508	−1.223	0.433
3. Actual X Inferred	0.080	0.049	0.626	1.635	0.171
**Relationship Quality - Adolescent (RQ-A)**					
**Perspective Taking** Step 1					
1. Preferred Ratings	−0.132	0.064	−0.163	−2.065	0.123
2. Inferred Ratings	0.243	0.065	0.295	3.730	<0.001
3. Actual Ratings	0.064	0.066	0.076	0.964	0.510
Step 2					
1. Preferred X Inferred	0.047	0.043	0.398	1.095	0.412
2. Preferred X Actual	0.060	0.037	0.537	1.591	0.342
3. Actual X Inferred	−0.026	0.042	−0.205	−0.632	0.564
**Perspective Getting** Step 1					
1. Preferred Ratings	0.107	0.051	0.158	2.105	0.037
2. Inferred Ratings	0.304	0.062	0.371	4.934	<0.001
3. Actual Ratings	0.110	0.072	0.115	1.526	0.129
Step 2					
1. Preferred X Inferred	−0.010	0.031	−0.093	−0.310	0.757
2. Preferred X Actual	0.044	041	460	1.065	0.433
3. Actual X Inferred	−0.042	0.042	−0.391	−0.982	0.328
**Adolescent’s Life Satisfaction**					
**Perspective-Taking** Step 1					
1. Preferred Ratings	−0.061	0.039	−0.123	−1.550	0.185
2. Inferred Ratings	0.151	0.040	0.300	3.774	<0.001
3. Actual Ratings	0.021	0.041	0.041	0.518	0.605
Step 2					
1. Preferred X Inferred	−0.001	0.026	−0.019	−0.052	0.958
2. Preferred X Actual	0.017	0.023	0.247	0.725	0.470
3. Actual X Inferred	−0.038	0.026	−0.482	−1.472	0.429
**Perspective-Getting** Step 1					
1. Preferred Ratings	096	0.032	0.232	2.974	0.005
2. Inferred Ratings	0.080	0.039	0.160	2.049	0.042
3. Actual Ratings	0.101	0.046	0.174	2.219	0.075
Step 2					
1. Preferred X Inferred	−0.011	0.020	−0.175	−0.565	0.757
2. Preferred X Actual	−0.018	0.026	−0.310	−0.693	0.490
3. Actual X Inferred	−0.042	0.027	−0.656	−1.588	0.171

### Associations between Perceived Understanding and Preferred, Inferred, and Actual Strategies

A hierarchical regression analysis was conducted, with preferred, inferred, and actual strategy and the interactions between them on perceived understanding.

**Fig. 3:**
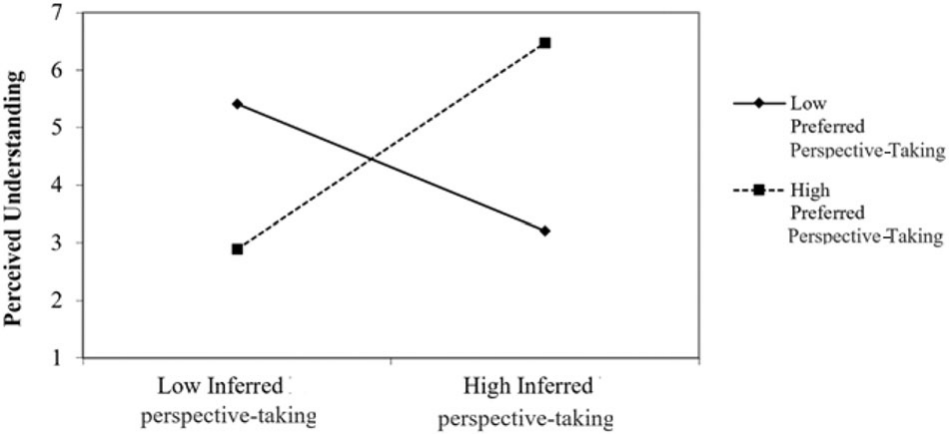
Perceived understanding for high vs. low levels of preferred perspective-taking, as a function of levels of inferred perspective-taking. Note. The interaction was plotted as recommended by Aiken & West (1991)—one SD above the mean of the inferred perspective-taking ratings, and one SD below the mean, in high- vs. low-ratings preferred perspective-taking

For perspective-taking, the first step was significant: *F* (3146) = 9.82, *p* < 0.001, *r*^2^ = . 17. Inferred perspective-taking significantly contributed to the model (*t* = 5.00, *β* = 0.38, *p* < 0.001)—the more participants inferred their parents used perspective-taking, the higher their ratings of perceived understanding. The second step was also significant: *F*(6143) = 7.22, *p* < 0.001, *r*^2^ = 0.23. The interaction between preferred and inferred perspective-taking was significant (*t* = 3.33, β = 1.14, *p* = 0.003). As Fig. 3 shows, when preferred perspective-taking was low, inferred perspective-taking was not associated with perceived understanding. However, when levels of preferred perspective-taking were average and high (1 SD above the average), perceived understanding was positively related to higher inferred perspective-taking.

For perspective-getting, the first step was significant: *F* (3145) = 14.46, *p* < 0.001, *r*^2^ = 0.23. The main effects of preferred and inferred perspective-getting were significant (preferred perspective-getting, *t* = 3.26, *β* 0.23, *p* = 0.003; inferred perspective-getting, *t* = 5.17, *β* = 0.37, *p* < 0.001), such that higher ratings of preferred and inferred perspective-getting were related to higher perceived accuracy. The second step was also significant: *F* (6142) = 8.12, *p* < 0.001, *r*^2^ = 0.25. None of the interactions were significant.

### Associations between Relationship Quality (from the Adolescent’s Perspective) and Preferred, Inferred, and Actual Strategies

A hierarchical regression analysis was conducted, with preferred, inferred, and actual strategy and the interactions between them on the mean ratings of relationship quality from the adolescents’ perspective (α = 0.90). For perspective-taking, the first step was significant: *F* (3146) = 6.10, *p* < 0.001, *r*^2^ = 0.11. Preferred perspective-taking significantly contributed to the model (*t* = −2.06, *β* = −0.16, *p* = 0.04)—the more adolescents preferred perspective-taking, the lower their ratings of relationship quality. Inferred perspective-taking was also a significant predictor (*t* = 3.73, *β* = 0.29, *p* < 0.001). The more participants inferred their parents used perspective-taking, the higher their ratings of relationship quality. Actual perspective-taking made no significant contribution to the model (*t* = 0.96, *β* = 0.07, *p* = 0.34). The second step was also significant: *F*(6143) = 3.71, *p* = 0.002, *r*^2^ = 0.13. However, none of the interactions contributed significantly to the model.

For perspective-getting, the first step was significant: *F* (3145) = 10.86, *p* < 0.001, *r*^2^ = 0.18. The role of preferred perspective-getting significantly contributed to the model (*t* = 2.10, *β* = 0.16, *p* = 0.03)— the more adolescents preferred perspective-getting, the higher their ratings of relationship quality. Inferred perspective-getting was also a significant predictor (*t* = 4.93, *β* = 0.37, *p* < 0.001). The more participants inferred their parents use perspective getting, the higher their ratings of relationship quality. Actual perspective-getting made no significant contribution to the model (*t* = 0.15, *β* = 0.11, *p* = 0.13). The second step was also significant: *F*(6142) = 5.76, *p* < 0.001, *r*^2^ = 0.19. However, none of the interactions contributed significantly to the model.

### Associations between Adolescent’s Life Satisfaction and Preferred, Inferred, and Actual Strategies

A hierarchical regression analysis was conducted, with preferred, inferred, and actual strategy and the interactions between them on the mean ratings of the life satisfaction scale (α = 0.79).

For perspective-taking, the first step was significant: *F* (3146) = 5.44, *p* = 0.001, *r*^2^ = 0.10. Inferred perspective-taking significantly contributed to the model (*t* = 3.77, *β* = 0.30, *p* < 0.001): the more participants inferred their parents used perspective-taking, the higher their satisfaction with life. The effects of preferred perspective-taking and actual perspective-taking were not significant (*t* = −1.55, *β* = −0.12, *p* = 0.12*; t* = 0.51, *β* = 0.04, *p* = 0.60, respectively). The second step was also significant: *F*(6143) = 3.15, *p* = 0.006, *r*^2^ = 0.12. However, none of the interactions contributed significantly to the model.

For perspective-getting, the first step was significant: *F* (3145) = 6.37, *p* < 0.001, *r*^2^ = 0.11. Both preferred and inferred perspective-getting made significant contributions to the model (preferred perspective-getting: *t* = 2.97, *β* = 0.23, *p* = 0.005; inferred perspective-getting: *t* = 2.05, *β* = 0.16, *p* = 0.042), such that the more participants preferred and inferred their parents use perspective-getting, the higher their ratings of life satisfaction. The second step was also significant: *F* (6142) = 3.70, *p* = 0.002, *r*^2^ = 0.13. None of the interactions were significant. Repeating the regression analysis with SPANE (positive and negative feelings, separately) as DV did not reveal significant results; see Supplementary Materials.

## Discussion

Research on adolescent-parent communication has highlighted the importance of perceived understanding for adolescents’ well-being and satisfaction with their relationship with their parents. However, little is known about how adolescents prefer their parents to try to accurately understand them. This study is an initial step in closing this gap by examining the mind-perception strategies adolescents wish their parents would use to accurately understand their feelings. The current study compared adolescents’ preferences with their perceptions of the strategies their parents use and with the parents’ reports of the strategies they actually use. The study focused on perspective-taking and perspective-getting as two central mind-perception strategies that were previously tested (Eyal et al., 2018) and that were also found to be the ones that adolescents in the current study preferred most.

The results revealed that adolescents mainly wished their parents would use perspective-taking. This preference can be understood in light of several psychological and relational dynamics. First, it may reflect the common and naïve belief in the power of taking the other’s perspective as an effective means to improve understanding, despite previous research indicating that perspective-taking is ineffective for accurate mind perception (Eyal et al., 2018). Second, this finding might also imply the adolescents’ preferences for mind-perception strategies are guided not only by the desire to be accurately understood (as highlighted in the study’s instructions) but also by other motivations, such as the need for autonomy and separation (e.g., Koepke & Denissen, 2012).

Another possible interpretation of the high preference for perspective-taking in the unique context of adolescent-parent relationship may be the adolescents’ expectation that their parents “put themselves in their shoes” as an egocentric desire to be understood without the need for direct communication (as required in perspective-getting). A recent qualitative study explored young people’s (aged 16–24) experiences and conceptualization of “feeling (mis)understood” in the context of help-seeking behavior (Cunningham et al., 2024). A central theme that emerged in this study was that the young participants wished adults would proactively support them, by making a significant effort to understand how they want to communicate, even if they perceived them to be less communicative and their behavior as challenging. Adolescents’ preference for perspective-taking as the main strategy in the present study may echo this pattern, suggesting that to feel accurately understood, adolescents expect their parents to make a special effort by trying to take their perspective, while they themselves remain somewhat passive in the interaction.

The discrepancies between preferred, inferred and actual (reported) strategies reveal important insights into the different means that parents and adolescents rely on to achieve accurate understanding. Perspective-taking showed the largest gap between the percentage of adolescents who preferred their parents would use it (42%) and those who inferred their parents used it (9%). A much smaller gap emerged between preferred (38%) and inferred (24%) perspective-getting, potentially because perspective-taking, unlike perspective getting, cannot be observed.

From the parents’ perspective, perspective getting and observed behavior were rated as the strategies they used the most, implying that parents focus on getting direct information by observing their children’s behavior or simply asking them about their feelings. This finding may reflect the parents’ overall tendency to perceive their communication with their adolescents as effortful and open) e.g., Kapetanovic & Boson, 2022; Xiao et al., 2011). Furthermore, using more direct strategies, such as perspective-getting, appears to be a reasonable choice for parents navigating the delicate balance of closeness and separation that is essential to their adolescents’ development. Indeed, studies on adolescents’ information management revealed greater disclosure from adolescents when their parents directly asked them questions (Keijsers et al., 2010; Soenens et al., 2006).

Comparing the adolescents’ inferences about the strategies their parents use with the strategies the parents report using, the data reveal the adolescents are relatively accurate. Specifically, similarly to the parents’ report of the strategies they use, adolescents inferred their parents mostly use perspective-getting, previous knowledge, and observed behavior and less so perspective-taking and projection. This correspondence between the adolescents’ and parents’ perceptions suggests adolescents are quite sensitive to and aware of their parents’ behavior and the strategies their parents use to understand them.

Adolescents’ perceptions and preferences regarding their parents’ use of perspective-getting and perspective-taking emerge as key predictors of their life satisfaction, perceived understanding, and relationship quality. The findings draw a consistent positive link between preferred and inferred perspective-getting and adolescents’ perceived understanding, relationship quality, and life satisfaction. These associations may point to the adolescents’ understanding of the benefits of perspective-getting for mental health and positive relationships with close others, as was previously found among adults in various interpersonal contexts (e.g., interpersonal accuracy, Eyal et al., 2018; attitudes toward outgroup members, Kalla & Broockman, 2023). Perspective-getting involves listening and self-disclosure, which are important features of interpersonal communication (Vijayakumar & Pfeifer, 2020; Yavuz& Celik, 2017). The presence of these features in the adolescents’ communication with their parents was found to be positively related to adolescents’ well-being and relationship satisfaction (e.g., Elsharnouby & Dost-Gözkan, 2020; Weinstein et al., 2021).

Adolescents’ perceived understanding, relationship quality, and life satisfaction were also positively associated with inferred perspective-taking, suggesting adolescents’ perceptions of their parents’ effort to understand them are highly important for their mental health and positive experience of their relationships with close others. This positive link can also be understood in light of the developmental stage of mid to late adolescence (compared with early adolescence). This period is characterized by a decrease in parental control, detachment, and the intensity of conflict between parents and children (Keijsers & Poulin, 2013; Smetana & Rote, 2019), which support the adolescents’ need for independence and autonomy as the main age-related task (Koepke & Denissen, 2012). Thus, adolescents’ inferred perspective-taking and its link to perceived understanding and relationship quality may reflect the match between adolescents’ natural need for autonomy and separation during this period and parents who are perceived to respect this need (i.e., by trying to understand their child in an indirect way).

Perceived understanding, relationship quality, and life satisfaction were associated with adolescents’ preferences and inferences regarding their parents’ behavior, but not with the gap between adolescents’ perceptions (preferences and inferences) and their parents’ reported actual strategy. These findings highlight adolescents’ experience and their idiosyncratic perceptions of their parents as main factors in explaining their mental health and relationship with others. Although this pattern seems surprising, it aligns with the findings of previous research on family communication, according to which adolescents’ perceptions of the family communication distinctively predict their mental health beyond their parents’ reports (Xiao et al., 2011), suggesting youth perceptions are more important than parents’ reports in predicting their psychosocial adjustment.

### Study Limitations and Future Directions

A possible limitation for the generalizability of the findings is the study’s focus on mid to late adolescence. This period is characterized by a decrease in parental control, detachment, and the intensity of conflict between parents and children (e.g., Keijsers & Poulin, 2013; Smetana & Rote, 2019), which support the adolescents’ need for independence and autonomy as the main age-related task (Koepke & Denissen, 2012). The results can be understood in light of this developmental stage, suggesting that adolescents’ preference for perspective-taking may reflect their need for separation. From the parents’ perspective, relying on perspective-getting might be perceived as less controlling (i.e., gathering direct information rather than imposing their own viewpoint). Future research could extend this examination to early adolescents, enabling a broader investigation of strategy preferences and use in alignment with different developmental stages.

Another limitation of this study is that participants were intentionally directed to think about a situation in which they felt unpleasant feelings, because such situations highlight the importance of accurate understanding by close others (Martin et al., 2018). However, sharing unpleasant experiences might be more difficult for adolescents than sharing pleasant ones, thus enhancing adolescents’ preference that their parents use perspective-taking over other, more direct and accurate mind-perception strategies such as perspective-getting. Future research is needed to explore the generalization of the current study’s findings when considering various life events, or during actual parent-adolescent conversations.

Finally, the demographic composition of the parents’ sample, is also a limitation of this study. The sample included a high percentage of mothers, which prevented exploration of possible differences between adolescents’ preferences for mind-perception strategies from mothers relative to fathers. In addition, demographic characteristics of the study sample in terms of socioeconomic status or cultural background were collected. Future research should test whether demographic factors interact with adolescents’ preferences and parents’ use of mind-perception strategies.

## Conclusion

Previous research has highlighted adolescent-parent communication—and perceived understanding in particular—as crucial to adolescents’ socioemotional outcomes and their relationship satisfaction with their parents. Yet, the means by which adolescents prefer their parents to accurately understand them has not been examined. The current study took a first step in exploring adolescents’ preferences for and inferences of the mind-perception strategies their parents use, and their parents’ reports on the strategies they use when trying to understand their child’s feelings. The results revealed a gap between the adolescents’ expectation that their parents will put themselves in their shoes and understand them without words and the parents’ attempt to communicate with their child in order to understand them. The findings also emphasize the role of the adolescent’s perceptions, regardless of the parent’s point of view, in understanding their life satisfaction and their satisfaction from the relationship with their parents.

## Supplementary information

Supplementary Materials
