# Supplementary material for: Adolescents’ Preferred and Inferred Strategies for Being Accurately Understood by Their Parents
Source: J Youth Adolesc. 2025 May 12;54(9):2301–12. doi: 10.1007/s10964-025-02193-w (PMC12420688; doi:10.1007/s10964-025-02193-w)
Supplement: Supplementary file 1 — Supplementary Materials [file 10964_2025_2193_MOESM1_ESM.docx]

**Adolescents’ Preferred and Inferred Strategies for Being Accurately Understood by Their Parents**

**Supplementary Materials**

The following are (1) regression analyses results for observed behavior, previous knowledge and projection (2) detailed analyses of the moderation model conducted to interpret the interaction effects described in the manuscript; and (3) analyses of additional variables that were assessed, but are not directly related to the main purpose of the study (i.e. parents' confidence and their relationship quality).

**Table 1.**

*Linear Hierarchical Regression Analyses for Variables Predicting Perceived Understanding, Life satisfaction and Relationship Quality from the adolescent's perspective.*

*NOTE. Alpha was corrected for multiple comparisons using the Benjamini-Hochberg adjustment (Benjamini & Hochberg, 1995).*

|  | | **B** | | **SE** | **β** | | **t** | | **BHp** | |
| --- | --- | --- | --- | --- | --- | --- | --- | --- | --- | --- |
| **Perceived Understanding** | | | | | | | | | | |
| **Previous Knowledge**  Step 1 | | | | | | | | | | |
| 1. Preferred Ratings | | .146 | | .076 | .153 | | 1.916 | | .057 | |
| 1. Inferred Ratings | | .238 | | .079 | .239 | | 2.998 | | .005 | |
| 1. Actual Ratings | | -.116 | | .095 | -.096 | | -1.215 | | .366 | |
| Step 2 | | | | | | | | | | |
| 1. Preferred X Inferred | | .066 | | .047 | .506 | | 1.417 | | .477 | |
| 1. Preferred X Actual | | -.035 | | .055 | -.258 | | -.636 | | .526 | |
| 1. Actual X Inferred | | .147 | | .070 | 1.082 | | 2.103 | | .089 | |
| **Observed Behavior**  Step 1 | | | | | | | | | | |
| 1. Preferred Ratings | | .126 | | .061 | .150 | | 2.059 | | .041 | |
| 1. Inferred Ratings | | .407 | | .066 | .445 | | 6.127 | | <.001 | |
| 1. Actual Ratings | | .128 | | .116 | .080 | | 1.103 | | .408 | |
| Step 2 | | | | | | | | | | |
| 1. Preferred X Inferred | | .049 | | .038 | .408 | | 1.305 | | .294 | |
| 1. Preferred X Actual | | -.011 | | .067 | -.081 | | -.158 | | .875 | |
| 1. Actual X Inferred | | .005 | | .070 | .033 | | .065 | | .948 | |
| **Projection***   - The Step 1 | | | | | | | | | | |
| 1. Preferred Ratings | | .092 | | .069 | .111 | | 1.330 | | .558 | |
| 1. Inferred Ratings | | .101 | | .073 | .117 | | 1.392 | | .249 | |
| 1. Actual Ratings | | -.125 | | .082 | -.128 | | -1.534 | | .191 | |
| Step 2 | | | | | | | | | | |
| 1. Preferred X Inferred | | .126 | | .036 | .992 | | 3.496 | | < .001 | |
| 1. Preferred X Actual | | -.020 | | .046 | -.157 | | -.444 | | .783 | |
| 1. Actual X Inferred | | -.071 | | .048 | -.507 | | -1.486 | | .420 | |
| **Relationship Quality from the adolescent's perspective (RQ-A)** | | | | | | | | | | |
| **Previous Knowledge**  Step 1 | | | | | | | | | | |
| 1. Preferred Ratings | | .165 | | .060 | .208 | | 2.766 | | .018 | |
| 1. Inferred Ratings | | .305 | | .062 | .366 | | 4.889 | | <.001 | |
| 1. Actual Ratings | | -.080 | | .075 | -.079 | | -1.066 | | .366 | |
| Step 2 | | | | | | | | | | |
| 1. Preferred X Inferred | | -.017 | | .037 | -.157 | | -.464 | | .743 | |
| 1. Preferred X Actual | | -.049 | | .043 | -.434 | | -1.132 | | .408 | |
| 1. Actual X Inferred | | .105 | | .055 | .925 | | 1.904 | | .089 | |
| **Observed Behavior**  Step 1 | | | | | | | | | | |
| 1. Preferred Ratings | | .126 | | .054 | .179 | | 2.313 | | .041 | |
| 1. Inferred Ratings | | .226 | | .059 | .296 | | 3.825 | | <.001 | |
| 1. Actual Ratings | | .119 | | .103 | .089 | | 1.155 | | .408 | |
| Step 2 | | | | | | | | | | |
| 1. Preferred X Inferred | | .023 | | .034 | .228 | | .684 | | .495 | |
| 1. Preferred X Actual | | .017 | | .059 | .160 | | .290 | | .875 | |
| 1. Actual X Inferred | | -.032 | | .062 | -.280 | | -.519 | | .906 | |
| **Projection***  Step 1 | | | | | | | | | | |
| 1. Preferred Ratings | | .026 | | .058 | .037 | | .447 | | .655 | |
| 1. Inferred Ratings | | .117 | | .061 | .161 | | 1.917 | | .171 | |
| 1. Actual Ratings | | -.127 | | .068 | -1.55 | | -1.863 | | .191 | |
| Step 2 | | | | | | | | | | |
| 1. Preferred X Inferred | | .066 | | .031 | .621 | | 2.158 | | .049 | |
| 1. Preferred X Actual | | -.084 | | .039 | -.778 | | -2.171 | | .096 | |
| 1. Actual X Inferred | | .002 | | .041 | .017 | | .050 | | .960 | |
| **Adolescent's Life Satisfaction** | | | | | | | | | | |
| **Previous Knowledge**  Step 1 | | | | | | | | | | |
| 1. Preferred Ratings | | .084 | | .039 | .174 | | 2.167 | | .048 | |
| 1. Inferred Ratings | | .110 | | .041 | .217 | | 2.714 | | .008 | |
| 1. Actual Ratings | | -.044 | | .049 | -.072 | | -.907 | | .366 | |
| Step 2 | | | | | | | | | | |
| 1. Preferred X Inferred | | -.008 | | .024 | -.119 | | -.329 | | .742 | |
| 1. Preferred X Actual | | -.031 | | .028 | -.453 | | -1.102 | | .272 | |
| 1. Actual X Inferred | | .061 | | .036 | .888 | | 1.707 | | .090 | |
| **Observed Behavior**  Step 1 | | | | | | | | | | |
| 1. Preferred Ratings | | .074 | | .034 | .174 | | 2.196 | | .041 | |
| 1. Inferred Ratings | | .114 | | .037 | .246 | | 3.118 | | .002 | |
| 1. Actual Ratings | | .019 | | .064 | .023 | | .294 | | .769 | |
| Step 2 | | | | | | | | | | |
| 1. Preferred X Inferred | | .027 | | .021 | .439 | | 1.299 | | .294 | |
| 1. Preferred X Actual | | -.024 | | .037 | -.363 | | -.651 | | .875 | |
| 1. Actual X Inferred | | .037 | | .038 | .525 | | .963 | | .906 | |
| **Projection***  *For Projection, both steps of the model were not significant  Step 1 | | | | | | | | | | |
| Preferred Ratings | .029 | | .036 | | | .068 | | .798 | | .639 |
| Inferred Ratings | .042 | | .038 | | .094 | | | 1.106 | | .271 |
| Actual Ratings -.016 .042 -.032 -.373 .709 | | | | | | | | | | |

| Step 2  Preferred X Inferred .012 .019 .180 .602 .548 |
| --- |
| Preferred X Actual -.007 .025 -.103 -.275 .783 |
| Actual X Inferred -.017 .026 -.236 -.655 .769 |

**Perceived Understanding: Moderation Analysis**

As reported in the manuscript, the regression analysis for perceived understanding revealed a significant interaction between preferred and inferred perspective-taking (*t* = 3.33, β = 1.14, *p* = .001). The role of inferred perspective-taking in predicting perceived understanding at the various levels of preferred perspective-taking (as a moderator), was examined using Hayes’ PROCESS Macro for SPSS (Hayes, 2013) Model 1.

Results indicated that under low levels of preferred perspective-taking (1 SD below the mean), inferred perspective-taking was not significantly related to perceived understanding, (*t* = 1.05, *p* = .29, 95% CI [-.101, .332]). However, under average and high (1 SD above the preferred perspective-taking mean) levels of preferred perspective-taking, inferred perspective-taking significantly predicted perceived understanding (*t* = 5.89, *p* < .001, 95% CI .291, .584]; *t* = 6.27, *p* < .001, 95% CI [.410, .787], respectively).

**Parent's Relationship Quality and confidence**

The following analyses were conducted to explore the parent's point of view, namely, parent's relationship quality and confidence (i.e. the extent to which they think they accurately understand their child’s feelings). To that aim, the same regression model as in the main manuscript was conducted, with parent's relationship quality and confidence as the DV (in separate analyses) as a function of the five strategies. As in the manuscript, the focus was on the main two strategies, namely, perspective-taking and perspective-getting from each perspective, and the interaction between them. The detailed results are reported in Table 2.

***Predicting relationship quality (the parent's perspective) from preferred, inferred and actual strategies***

The parent's relationship quality index for each participant was conducted as the average of the four items (α = .81).

For perspective taking, the first step was significant: *F* (3,146) = 3.38, *p* = .02, $r^{2}$ $r^{2}$=. 06. Actual perspective-taking significantly contributed to the model (*t* = 2.28, *β* = .18, *p* = .02). The more the parent reported using perspective-taking the higher their ratings of relationship quality. The effect of preferred and inferred perspective-taking were not significant (*t* = 1.28, *β* = .10, *p* = .20; *t* = -1.92, *β* = -.15, *p* = .112). The second step was also significant: *F*(6,143) = 2.67, *p* = .017, $r^{2}$= .10. The interaction between preferred perspective-taking and actual perspective-taking was significant (*t* = 2.28, *β* = .78, *p* = .02), see Fig. 1. To better understand the interaction effect, the role of actual perspective-taking in predicting parents' relationship quality at the various levels of preferred perspective-taking (as a moderator) was examined, using Hayes’ PROCESS Macro for SPSS (Hayes, 2013) Model 1.

Results indicated that under low levels of preferred perspective-taking (1 SD below the mean), actual perspective-taking did not significantly predicted parents' relationship quality, (*t* = .77 , *p* = .44, 95% CI [-.067, .155]). However, under average and high (1 SD above the preferred perspective-taking mean) levels of preferred perspective-taking, actual perspective-taking significantly predicted parents' relationship quality (*t* = 3.25, *p* = .001, 95% CI [.066, .272]; *t* = 3.42, *p* < .001, 95% CI [.098, .366], respectively). This pattern may reflect the parent's response to disparity between the high expectation of their adolescent, and their actual response to the this need.

***
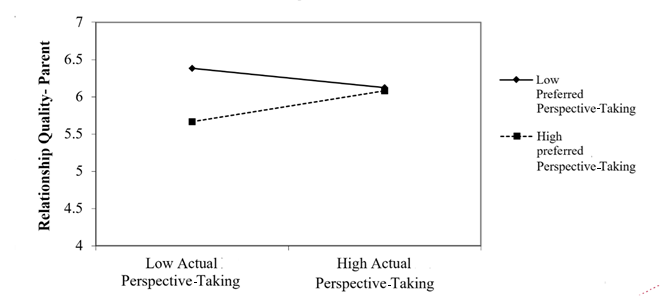
***

***Fig. 1*** *The parent's relationship quality for high vs. low levels of preferred perspective-taking, as a function of levels of actual perspective-taking*

*Note. The interaction was plotted as recommended by Aiken and West (1991)—one SD above the mean of actual perspective-taking, and one SD below the mean in high vs. low ratings of preferred perspective-taking*

The subsequent analysis focused on whether relationship quality from the parent's perspective was predicted by perceptions of perspective-getting. The first step was significant: *F* (3,145) = 10.00, *p* < .001, $r^{2}$ $r^{2}$=. 17. Only the effect of actual perspective-getting significantly contributed to the model (*t* = 5.12, *β* =.39, *p* < .001)—the more parents reported using perspective-getting the higher were their ratings of relationship quality. Preferred and inferred perspective-getting made no significant contribution to the model (preferred perspective-getting: *t* = -1.23, *β* = -.09, *p* = .44; Inferred perspective-getting: *t* = 1.27, *β* = .09, *p* = .24). The second step was also significant: *F* (6,142) = 7.03, *p* < .001, $r^{2}$= .23. Only the interaction between inferred perspective-getting and actual perspective-getting was significant (*t* = -2.98, β = -1.16, *p*  = .006), see Fig. 2.

Results of a moderation model^^[[1]](#footnote-1)^^ indicate that under high level of inferred perspective-getting (1 SD above the mean), actual perspective-getting did not significantly predict parents' relationship quality, (*t* = 1.32, *p* = .19, 95% CI [-.048, .240]). However, under low (1 SD below the inferred perspective-getting mean) and average levels of inferred perspective-getting, actual perspective-getting significantly predicted RQ-P (*t* = 5.76, *p* < .001, 95% CI [.297, .608]; *t* = 5.52, p < .001, 95% CI [.176, .373], respectively).

As can be seen in Figure 2, when parents reported using perspective-getting to a large extent, their ratings of relationship quality were high, irrespective of the adolescents’ inferred perspective-getting. However, when the parents reported using perspective-getting to a small extent, their ratings of relationship quality were high only when the adolescents’ inferred perspective-getting was high. Relationship quality was lowest when both the parent did not report using perspective-getting and the adolescents inferred they did not use perspective-getting.

***
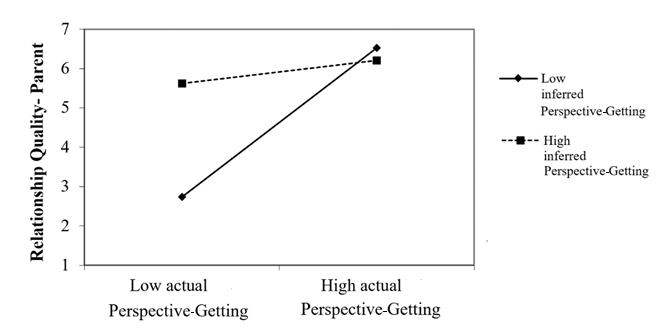
***

***Fig. 2*** *The parent's relationship quality for high vs. low levels of inferred perspective-getting, as a*

*function of levels of actual perspective-getting*

***Note.*** The interaction was plotted as recommended by Aiken and West (1991)—one SD above the mean of the actual perspective-getting ratings, and one SD below the mean, in high vs. low ratings inferred perspective-getting***.***

***Predicting the parent's confidence from preferred, inferred and actual strategies***

The parent’s confidence (i.e., the extent to which they reported they accurately understand their child’s feelings) as a function of perceptions of perspective-taking and perspective-getting (separately) was examined, using the same hierarchical regression analysis.

The first step of the model, for perceptions of perspective-taking, was significant: *F* (3,146) = 4.86, *p* = .003, $r^{2}$ $r^{2}$=. 09. Only actual perspective-taking significantly contributed to the model (*t* = 3.16, *β* = .25, *p* = .004)—such that the more participants parents reported they use perspective-taking to higher extent, the higher were their confidence ratings. The second step was also significant: *F*(6,143) = 4.49, *p* < .001, $r^{2}$= .16. The interaction between preferred and actual perspective-taking significantly contributed to the model (*t* = 3.17, *β* = 1.05, *p* = .004), see Fig. 3. Results of the above moderation model (with parent's confidence as DV, actual perspective-taking as predictor and preferred perspective-taking as a moderator) indicated that under low level of preferred perspective-taking (1 SD above the mean), actual perspective-taking did not significantly predict parents' confidence, (*t* = 1.04, *p* = .129, 95% CI [-.064, .207]). However, under average and high (1 SD above the preferred perspective-taking mean) levels of preferred perspective-taking, actual perspective-taking significantly predicted parent's confidence (*t* = 4.55, *p* < .001, 95% CI [.164, .416]; *t* = 4.81, p < .001, 95% CI [.235, .563], respectively). This pattern is probably a reflection of the match between the adolescent's wish and their parent’s reported use of this strategy.

***
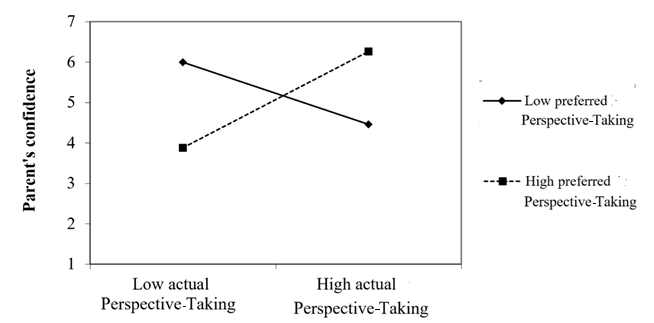
***

***Fig. 3*** *Parent's confidence* *for high vs. low levels of preferred perspective-taking, as a function of*

*levels of actual perspective-taking*

Note. The interaction was plotted, as recommended by Aiken and West (1991)—one SD above the mean of the preferred perspective-taking ratings, and one SD below the mean, in high vs. low ratings of actual perspective-taking

The subsequent analysis focused on whether the parent's confidence was predicted by perceptions of perspective-getting. The same hierarchal regression analysis was conducted with ratings of preferred, inferred and actual perspective-getting and their two-way interactions as predictors. The first step was significant: *F* (3,145) = 13.46, *p* < .001, $r^{2}$ $r^{2}$=. 22. Only actual perspective-getting significantly contributed to the model (*t* = 5.89, *β* =.43, *p* < .001)—such that higher ratings of perspective-getting as the actual strategy were related to higher confidence of the parent. The second step was also significant: *F*(6,142) = 8.20, *p* < .001, $r^{2}$= .26. The interaction between inferred and actual perspective-getting significantly contributed to the model (*t* = -2.25, *β* = -.86, *p* = .026). Results of a moderation model (with parent's confidence as a DV, inferred perspective getting as a predictor and actual perspective-getting as a moderator) revealed that under low levels of actual perspective-getting, inferred perspective-getting positively predicted parent's confidence (*t* = 2.95, *p* = .004, 95% CI [.076, .384]). However, under average and high levels of actual perspective-getting, inferred perspective-getting did not significantly predicted parent's confidence (*t* = 1.46, *p* = .15, 95% CI [-.027, .181]; *t* = -.09, p = .93, 95% CI [-.139, .127], respectively).

As can be seen in Fig. 4, when the parents’ reported using perspective-getting to a large extent, their ratings of confidence were high, irrespective of the adolescents’ inferred perspective-getting. However, when the parents reported using perspective-getting to a small extent, their ratings of confidence were high only when the adolescents’ inferred that perspective-getting was high. The parent's confidence was lowest when the parent did not report using perspective-getting and the adolescents inferred they did not use perspective-getting.

***
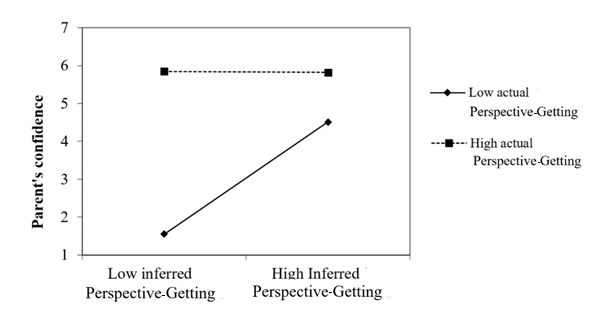
***

***Fig. 4*** *Parent's confidence* *for high vs. low levels of actual perspective-getting, as a function of levels of inferred perspective-getting*

*Note. The interaction was plotted (Fig. 7), as recommended by Aiken and West (1991)—one SD above the mean of the inferred perspective-getting ratings, and one SD below the mean, in high vs. low ratings actual perspective-getting*

The results of parents' variables (relationship quality and confidence) revealed an interesting pattern: While gaps between the adolescents’ and parents' perceptions did not predict adolescents’ outcomes (relationship quality, life satisfaction and perceived understanding); predictions of parents' relationship quality and confidence revealed the key role played by interactions between adolescents' preference and parents’ behavior (whether inferred by the adolescent or reported by the parent). Here too, perspective-taking and perspective-getting consistently demonstrated distinct patterns. Parents’ use of perspective taking predicted their relationship quality and confidence, only when adolescents' expectation from them to use perspective taking was high. This pattern highlights the match [disparity] between adolescents' preferences and parents' actual use of perspective-taking, in predicting parent-adolescent's relationship quality from the parent’s perspective, and the parent's confidence in their accurate understanding of their child’s feelings.

Looking at perspective-getting, only when parents reported lower use of perspective-getting, the gap between actual perspective-getting and the adolescents' perception of their parents (i.e. inferred perspective-getting) was a significant predictor of parent's relationship quality and confidence. This finding supports the positive role of higher use of perspective-getting in parents' confidence and relationship quality.

Finally, parents' ratings of their actual use of the two strategies were significant positive predictors for their relationship quality and for their confidence in their accuracy understanding of their child. It appears that merely the effort parents put in trying to understand their child (whether by using perspective-getting or perspective-taking) serve as positive predictors.

The fact that predictions of parents' relationship quality and adolescents' relationship quality yielded different patterns may emphasize the asymmetries of these relationship, and the divergent goals adolescents and parents hold within it. For example, while adolescents primarily seek for autonomy and self-identity, parents wish to maintain a sense of continuity and harmony in the relationship (also known as *the* *generational stake hypothesis*, Bengtson & Kuypers,1971). In the current study, parents' relationship quality was predicted by the interaction between parent's actual strategy and their adolescents' inferences or preferences, a pattern that may relate to the greater attention parents devote to the communication process, while adolescents' relationship quality was mostly linked to their own perspective.

**Table 2.**

*Linear Hierarchical Regression Analyses for Variables Predicting Relationship Quality from the Parent's perspective (*relationship quality *-parent), and the parent's confidence*

*NOTE. Alpha was corrected for multiple comparisons using the Benjamini-Hochberg adjustment (Benjamini & Hochberg, 1995).*

| \| \|  \| **B** \| **SE** \| **β** \| **t** \| **BHp** \| \| --- \| --- \| --- \| --- \| --- \| --- \| \| \| \| \| \| \| \| --- \| --- \| --- \| --- \| --- \| --- \| --- \| --- \| --- \| --- \| --- \| --- \| \| **Relationship Quality -Parent (RQ-P)** \| \| \| \| \| \| \| **Perspective-Taking**  Step 1 \| \| \| \| \| \| \| 1. Preferred Ratings \| -.089 \| .046 \| -.156 \| -1.927 \| .112 \| \| 1. Inferred Ratings \| .060 \| .047 \| .104 \| 1.278 \| .203 \| \| 1. Actual Ratings \| .110 \| .048 \| .185 \| 2.288 \| .024 \| \| Step 2 \| \| \| \| \| \| \| 1. Preferred X Inferred \| -.018 \| .031 \| -.211 \| -.570 \| .570 \| \| 1. Preferred X Actual \| .061 \| .027 \| .786 \| 2.283 \| .024 \| \| 1. Actual X Inferred \| -.013 \| .030 \| -.148 \| -.447 \| .655 \| \| **Perspective-Getting**  Step 1 \| \| \| \| \| \| \| 1. Preferred Ratings \| -.044 \| .036 \| -.093 \| -1.228 \| .442 \| \| 1. Inferred Ratings \| .051 \| .043 \| .089 \| 1.172 \| .243 \| \| 1. Actual Ratings \| .262 \| .051 \| .389 \| 5.122 \| <.001 \| \| Step 2 \| \| \| \| \| \| \| 1. Preferred X Inferred \| .002 \| .021 \| .029 \| .098 \| .922 \| \| 1. Preferred X Actual \| .035 \| .028 \| .516 \| 1.226 \| .444 \| \| 1. Actual X Inferred \| -.087 \| .029 \| -1.164 \| -2.986 \| .006 \|   **Previous Knowledge**  Step 1 | | | | | |
| --- | --- | --- | --- | --- | --- | --- | --- | --- | --- | --- | --- | --- | --- | --- | --- | --- | --- | --- | --- | --- | --- | --- | --- | --- | --- | --- | --- | --- | --- | --- | --- | --- | --- | --- | --- | --- | --- | --- | --- | --- | --- | --- | --- | --- | --- | --- | --- | --- | --- | --- | --- | --- | --- | --- | --- | --- | --- | --- | --- | --- | --- | --- | --- | --- | --- | --- | --- | --- | --- | --- | --- | --- | --- | --- | --- | --- | --- | --- | --- | --- | --- | --- | --- | --- | --- | --- | --- | --- | --- | --- | --- | --- | --- | --- | --- | --- | --- | --- | --- | --- | --- | --- | --- | --- | --- | --- | --- | --- | --- | --- | --- | --- | --- | --- | --- | --- | --- | --- | --- |
| 1. Preferred Ratings | .006 | .044 | .011 | .144 | .886 |
| 1. Inferred Ratings | .172 | .046 | .294 | 3.732 | <.001 |
| 1. Actual Ratings | .125 | .055 | .177 | 2.266 | .050 |
| Step 2 | | | | | |
| 1. Preferred X Inferred | .019 | .027 | .252 | .701 | .484 |
| 1. Preferred X Actual | .036 | .032 | .454 | 1.113 | .268 |
| 1. Actual X Inferred | .000 | .041 | -.005 | -.009 | .993 |
| **Observed Behavior**  Step 1 | | | | | |
| 1. Preferred Ratings | -.016 | .040 | -.032 | -.394 | .694 |
| 1. Inferred Ratings | .088 | .043 | .164 | 2.043 | .086 |
| 1. Actual Ratings | .179 | .075 | .191 | 2.373 | .019 |
| Step 2 | | | | | |
| 1. Preferred X Inferred | .074 | .024 | 1.043 | 3.118 | .002 |
| 1. Preferred X Actual | .048 | .042 | .626 | 1.136 | .516 |
| 1. Actual X Inferred | .050 | .044 | .614 | 1.139 | .514 |
| **Projection**  Step 1 | | | | | |
| 1. referred Ratings | -.086 | .040 | -.176 | -2.120 | .072 |
| 1. Inferred Ratings | .036 | .043 | .071 | .848 | .796 |
| 1. Actual Ratings | -.078 | .048 | -.136 | -1.641 | .103 |
| Step 2 | | | | | |
| 1. Preferred X Inferred | .028 | .021 | .374 | 1.309 | .193 |
| 1. Preferred X Actual | -.051 | .027 | -.674 | -1.894 | .060 |
| 1. Actual X Inferred | .064 | .028 | .779 | 2.269 | .050 |

| **Parent's Confidence**   \| **Perspective-Taking**  Step 1 \| \| \| \| \| \| \| --- \| --- \| --- \| --- \| --- \| --- \| \| 1. Preferred Ratings \| .038 \| .058 \| .053 \| .662 \| .509 \| \| 1. Inferred Ratings \| .086 \| .059 \| .117 \| 1.460 \| .203 \| \| 1. Actual Ratings \| .189 \| .060 \| .252 \| 3.160 \| .004 \| \| Step 2 \| \| \| \| \| \| \| 1. Preferred X Inferred \| .032 \| .038 \| .303 \| .844 \| .570 \| \| 1. Preferred X Actual \| .104 \| .033 \| 1.058 \| 3.170 \| .004 \| \| 1. Actual X Inferred \| .017 \| .037 \| .147 \| .461 \| .655 \| \| **Perspective-Getting**  Step 1 \| \| \| \| \| \| \| 1. Preferred Ratings \| .027 \| .044 \| .045 \| .610 \| .543 \| \| 1. Inferred Ratings \| .094 \| .053 \| .130 \| 1.762 \| .160 \| \| 1. Actual Ratings \| .370 \| .063 \| .435 \| 5.894 \| <.001 \| \| Step 2 \| \| \| \| \| \| \| 1. Preferred X Inferred \| .035 \| .026 \| .374 \| 1.310 \| .384 \| \| 1. Preferred X Actual \| .004 \| .035 \| .053 \| .127 \| .899 \| \| 1. Actual X Inferred \| -.084 \| .036 \| -.863 \| -2.256 \| .026 \| | | | | | |
| --- | --- | --- | --- | --- | --- | --- | --- | --- | --- | --- | --- | --- | --- | --- | --- | --- | --- | --- | --- | --- | --- | --- | --- | --- | --- | --- | --- | --- | --- | --- | --- | --- | --- | --- | --- | --- | --- | --- | --- | --- | --- | --- | --- | --- | --- | --- | --- | --- | --- | --- | --- | --- | --- | --- | --- | --- | --- | --- | --- | --- | --- | --- | --- | --- | --- | --- | --- | --- | --- | --- | --- | --- | --- | --- | --- | --- | --- | --- | --- | --- | --- | --- | --- | --- | --- | --- | --- | --- | --- | --- | --- | --- | --- | --- | --- | --- | --- | --- | --- | --- | --- |
| **Previous Knowledge**  Step 1 | | | | | |
| 1. Preferred Ratings | .071 | .057 | .101 | 1.246 | .430 |
| 1. Inferred Ratings | .150 | .060 | .203 | 2.520 | .013 |
| 1. Actual Ratings | .125 | .072 | .140 | 1.751 | .082 |
| Step 2 | | | | | |
| 1. Preferred X Inferred | .055 | .035 | .567 | 1.559 | .242 |
| 1. Preferred X Actual | .048 | .041 | .478 | 1.157 | .268 |
| 1. Actual X Inferred | .018 | .053 | .180 | .343 | .993 |
| **Observed Behavior**  Step 1 | | | | | |
| 1. Preferred Ratings | .057 | .050 | .091 | 1.148 | .506 |
| 1. Inferred Ratings | .062 | .054 | .092 | 1.161 | .248 |
| 1. Actual Ratings | .318 | .094 | .270 | 3.396 | <.001 |
| Step 2 | | | | | |
| 1. Preferred X Inferred | .116 | .029 | 1.291 | 3.965 | <.001 |
| 1. Preferred X Actual | -.017 | .051 | -.179 | -.335 | .738 |
| 1. Actual X Inferred | -.017 | .054 | -.164 | -.313 | .775 |
| **Projection***  Step 1 | | | | | |
| 1. Preferred Ratings | .015 | .052 | .024 | .286 | .775 |
| 1. Inferred Ratings | -.010 | .055 | -.016 | -.184 | .854 |
| 1. Actual Ratings | -.119 | .061 | -.163 | -1.948 | .103 |
| Step 2 | | | | | |
| 1. Preferred X Inferred | .062 | .027 | .655 | 2.312 | .044 |
| 1. Preferred X Actual | .072 | .034 | .750 | .2.122 | .060 |
| 1. Actual X Inferred | .011 | .036 | .102 | .300 | .765 |

* The first step of the model for projection was not significant

1. The same model as described above, using Hayes’ PROCESS Macro for SPSS, Model 1, with actual perspective getting as predictor, parent's relationship quality as DV, and inferred perspective-getting as a moderator [↑](#footnote-ref-1)
